# IJA: An Efficient Algorithm for Query Processing in Sensor Networks

**DOI:** 10.3390/s110201682

**Published:** 2011-01-28

**Authors:** Hyun Chang Lee, Young Jae Lee, Ji Hyang Lim, Dong Hwa Kim

**Affiliations:** 1 Division of Information and e-Commerce, Wonkwang University, Iksan, Korea; E-Mail: hclglory@wku.ac.kr; 2 Department of Multimedia, Jeonju University, Jeonju, Korea; 3 Department of Art Therapy, Daegu Cyber University, Daegu, Korea; E-Mail: possible@dcu.ac.kr; 4 Control Instrumentation Engineering Major, Hanbat National University, Daejeon, Korea; E-Mail: kimdh@hanbat.ac.kr

**Keywords:** sensor network communication cost, incremental algorithm, in-network query processing, wireless sensor networks

## Abstract

One of main features in sensor networks is the function that processes real time state information after gathering needed data from many domains. The component technologies consisting of each node called a sensor node that are including physical sensors, processors, actuators and power have advanced significantly over the last decade. Thanks to the advanced technology, over time sensor networks have been adopted in an all-round industry sensing physical phenomenon. However, sensor nodes in sensor networks are considerably constrained because with their energy and memory resources they have a very limited ability to process any information compared to conventional computer systems. Thus query processing over the nodes should be constrained because of their limitations. Due to the problems, the join operations in sensor networks are typically processed in a distributed manner over a set of nodes and have been studied. By way of example while simple queries, such as select and aggregate queries, in sensor networks have been addressed in the literature, the processing of join queries in sensor networks remains to be investigated. Therefore, in this paper, we propose and describe an Incremental Join Algorithm (IJA) in Sensor Networks to reduce the overhead caused by moving a join pair to the final join node or to minimize the communication cost that is the main consumer of the battery when processing the distributed queries in sensor networks environments. At the same time, the simulation result shows that the proposed IJA algorithm significantly reduces the number of bytes to be moved to join nodes compared to the popular synopsis join algorithm.

## 1. Introduction

Technological advances, decreasing production costs and increasing capabilities have made sensor networks suitable for many application fields such as various scientific and commercial applications including warehouse management, battlefield surveillance and environmental monitoring [1–4]. Thanks to the advanced technology, over time sensor networks have been adopted in an all-around industry. Gathering data to be aware of any states by using those sensors is achieved by modeling it as a distributed database where sensor readings are collected and processed using queries [5–7].

Especially, sensor node components of sensor networks obtain the state information from sensor device parts on those nodes and store those data. Accordingly, each sensor node in a sensor network is regarded as a distributed database system generating a data stream and has been studied as a sensor database [7]. In query processing of sensor networks, the join operation costs much in sensor networks for correlating sensor readings like distributed database environments [8]. Therefore, many researchers have studied reducing the cost in sensor environment [1].

In a sensor network environment, a query is issued for retrieving and gathering the real time state information. The form of a well-used query in a sensor network is using an SQL-like declarative language [8]. The collected data in a sensor network can be seen as one distributed relation over the sensor nodes, called the sensor relation. The query operations are also served restrictively because of the limitation of the environments. Further, most previous solutions either assume that nodes have sufficient memory to buffer the partition of the join relations assigned to them for processing, or that the amount of memory available at each node is known in advance and the assigned data partitions can be set accordingly [1]. Under these assumptions, it is hard to apply assumptions to real life.

Therefore, we consider the communication cost aspect and we propose an Incremental Join Algorithm (IJA) as an in-network join strategy which is an efficient join processing in sensor networks and minimizes communication cost. The IJA strategy is capable of reducing communication cost and utilizing data by gathering real-time state information from sensors which is one of sensor network features. In sensor network environments, it is hard to send all data stored at each node to server located in the center as we consider in the assumptions. Therefore it needs to be filtered whether data are sent or not. The problem in the previous studies is that the results processed in the previous steps would be ignored as a query happens. However, as a different point between the earlier studies and this paper, this algorithm for processing query uses the previous result and just sends the operations needed to be joined to the final node. Therefore assume previous join results are stored in temporary repository to process efficiently. To evaluate the performance, we compare the IJA strategy to the conventional algorithms including synopsis strategy. The remainder of the paper is structured as follows. In the next section, we describe typical join strategies in sensor networks including synopsis to compare. In Section 3, we introduce and explain an incremental join algorithm. And we analyze the performance and compare IJA to typical join strategy including synopsis algorithm. In Section 5, we conclude with future works.

## 2. Related Works

In sensor network, sensor nodes are formed by hundreds and hundreds of fixed nodes. Consequently, the value obtained periodically from sensor nodes is lack of expressing all the information about event or entity. It needs a join operation for that problem. The data stored at each sensor node forms a kind of table over all nodes, denoted R. To process a join query, we first have to decide which join queries are used. In this paper, we consider binary equi-join (BEJ). A BEJ query for sensor networks is defined as follows:

*Definition 1*

$$\begin{array}{c} \text{Given two sensor tables }R\left(A_{1},A_{2},\;\;...,\;\;A_{n}\right)\;\text{and }S\left(B_{1},B_{2},\;\;...,\;\;B_{n}\right),\;\text{a binary equi-join (BEJ)}\;\text{is}\\ R\infty_{\text{Ai}=\text{Bj}}S\;\;\;\;\;\left(\text{i }\in \;\;\left\{1,2,\;\;....,\;n\right\},\text{j }\in \;\;\left\{1,2,\;\;....,\;m\right\}\right) \end{array}$$

where A_i_ and B_j_ are two attributes of R and S respectively, which have the same domain.

Consider a sensor network covering a road network from [7]. Each sensor node can detect the IDs of vehicles in its close vicinity, and record the timestamps at which the vehicles are detected. Suppose N_R_ and N_S_ represent two sets of sensor nodes located at two regions of a road segment, Region1 and Region2, respectively. To gather the necessary data for determining the speeds of vehicles traveling between the two regions, the following join query can be expressed:

SELECT R.autoID, R.time, S.time
FROM R, S
WHERE R.location IN Region1 AND S.location IN Region2 AND R.autoID = S.autoID

**Figure 1.**
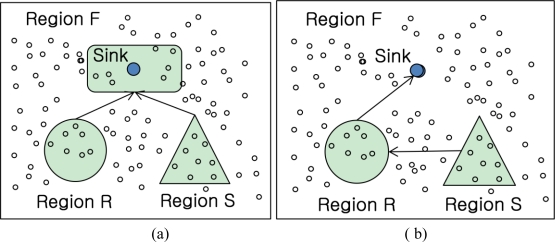

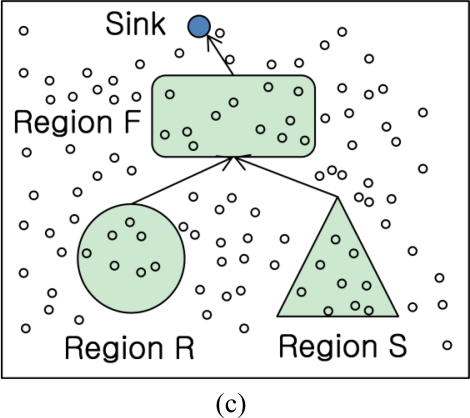
General join strategies. **(a)** Naïve join. **(b)** Sequential join. **(c)** Centroid join.

To evaluate the above query, sensor readings from Region1 and Region2 need to be collected and joined on the autoID attribute. Typical join strategies of sensor networks are classified into Naïve join, Sequential join and Centroid join according to the join location and shown in Figure 1 [9].

One of general join problems is a heavy communication cost to be transferred into among nodes and a lower join selectivity of query regarding overhead in communication. For instance, given there are two tables, R and S, pairs which is not participated in joining operation between R and S tables can be sent to another region F to join with another table. In the Naïve join algorithm in Figure 1(a), sensor nodes around the sink node in region F are join nodes N_F_ selected. Although the cost of routing join results to the sink node can be minimized, the each whole table in region R and S is routed to the sink.

In the Sequential join algorithm in Figure 1(b), it is minimized by routing the join results to the sink after performing the local join R_i_ ∞ S where R_i_ is the local table stored at node n_i_ in region R and S is the table in region S. In this algorithm, the problem is also that the whole table in region S is delivered to the nodes in region R. That makes communication cost be high.

As compared with the earlier algorithms, the Centroid algorithm in Figure 1(c) could also deliver the tables in each region into region F. In spite of that delivery, the nodes which are close to each region R and S in distance are selected to be joined and it could minimize the communication cost. However, the tables in each region are also needed to be routed into the join region F.

To solve or reduce the problems above, the synopsis strategy join was suggested. After reducing the number of pairs in R and S tables using synopsis to remove the rest pairs not to participate join operation, SNJ sends the pairs to join with others. The means of synopsis is an abstract of a table to process join operation. In addition, the size of the synopsis table is smaller than original table size. Therefore, each sensor creates its synopsis [9]. Synopsis strategy consists of 3 steps. First is synopsis join step. The second step is notification and third is final join operation.

The first join operation of synopsis is as follows: each node, n_i_ ∈ N_R_, stores the local table R_i_ which is one of local tables consisting of table R. Also each node n_i_ creates local synopsis S_i_ (R_i_) by extracting join attributes A_j_, and counting the frequency of the same value in the table. Synopsis join region N_L_ is selected to get a final join candidate pairs from joining table R and S synopsis. The synopsis join nodes after receiving synopsis from N_R_ and N_S_ synopsis process synopsis join operations.

The second step, a notification of synopsis join strategy, notifies final join candidate pairs to the N_R_ and N_S_ nodes. For this, synopsis join node n1 stores sensor ID of local synopsis originated. In the third step, each node of N_R_ or N_S_ notified from synopsis join nodes n_1_ sends join attribute v to final join node n_f_. The final join node n_f_ joins with R_v_ ∞ S_v_, and then sends the results to query sink node.

However, although the synopsis algorithm has contributed to reduce the communication cost, the algorithm only excludes duplicated data and a lot of data could be delivered as far as the table has various attribute values not to be duplicated. Therefore, we need to process as a unit of query and suggest an incremental join algorithm.

## 3. IJA: Incremental Join Algorithm

In this section, we propose an incremental join algorithm (IJA) to gather and process real-time state information which is one of the sensor network features. First, we describe general environment components including terms in next section [7,11] and the algorithm later.

### 3.1. General Environment

Suppose a sensor network consisting of *N* sensor nodes. We assume there are two virtual tables in the sensor network, *R* and *S*, containing sensor readings distributed in sensors. Each sensor reading is a pair with two mandatory attributes, timestamp and sensorID, indicating the time and the sensor at which the pair is generated. A sensor reading may contain other attributes that are measurements generated by a sensor or multiple sensors, e.g., temperature, autoID. We are interested in the evaluation of static one-shot binary equi-join queries in sensor networks. We assume that *R* and *S* are stored in two sets of sensor nodes *N_R_* and *N_S_* located in to distinct regions known as R and S respectively. A BEJ query can be issued from any sensor node called query sink, which is responsible for collecting the join result. A set of nodes is required to process the join collaboratively, referred to as join nodes.

When a join query is issued, a join node selection process is initiated to find a set of join nodes *N_F_* to perform the join. *R* pairs are routed to a join region *F* where the join nodes *N_F_* reside in. Each join node *n_f_* ∈*N_F_* stores a horizontal partition of the table *R*, denoted as *R_f_*. *S* pairs are transmitted to and broadcast in *F*. Each join node *n_f_* receives a copy of *S* and processes local join *R_f_* ∞ *S*. The query sink obtains the join results by collecting the partial join results at each *n_f_*.

The selection of *N_F_* is critical to the join performance. Join node selection involves selecting the number of nodes in *N_F_*, denoted by |*N_F_*|, and the location of the join region *F*. To avoid memory overflow, assuming *R* is evenly distributed in *N_F_*, |*N_F_*| should be at least |*R*|/*m*, where |*R*| denotes the number of pairs in *R* and *m* denotes the maximum number of *R* pairs a join node *n_f_* can store. For the rest, it carries out the experiments in the same condition with previous researches.

### 3.2. Incremental Join Algorithm Strategy

**Figure 2.**
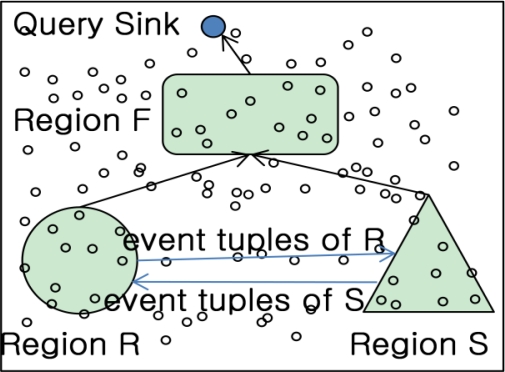
Incremental join algorithm.

Figure 2 shows the flow of IJA. The sink node in IJA is a node happened query and responsible for gathering the query results from each region similar to previous researches. The different point compared to the previous researches is that the result to be routed with each region is a kind of join results just participated in the query processing. For this, suppose that nodes in each region could know information of tables to be participating in join operations from query. Each node performs the local join operation based on the query information. For instance, the table *R* information in a query would join within *S* table existed in region S. In case of table *S*, it is relatively in the opposite direction. Therefore, we can get the information to perform local joins at each region through a query. If there is no longer local join operation in a region from query, then no further operation is needed to rout and deliver to the final node. In addition, owing to processing a unit of query, the communication cost could be remarkably reduced.

The steps for IJA are the following:

1. Send an event pair of *R*(or *S*) to other part. Only send the pair to *R*egio*n* S to make a semi table P_R_ (or P_S_) at the counterpart.
2. Perform join operation at each region to produce a semi table. Send the semi table to region F in case joining results exist.
3. Perform join operation with semi tables from R and S respectively. Send the join results to the query sink.
4. At the query sink node, the query can get the result within region F not to compute all of R and S computations.

The incremental join algorithm’s objective is to produce a smaller semi table by processing the join operation in the counter region because it needs to be decided whether the event pair is useful to join at the region or not. The rest of operations to process at the regions such as selecting a center location of the regions, routing protocol etc, is based on [7,13]. The number of the join nodes at semi table join region is decided by P_R_ and P_S_ arrived at N_H_ region. Therefore given memory m for a node, the node number at semi table region is as follows:

$$\left|N_{H}\right|=\left(\left|P_{R}\right|+\left|P_{S}\right|\right)/m$$

The different point with other algorithms is just sending the pair whenever a pair is occurred with insert or update operation. To compute the communication cost, |N_F_| is as follows:

$$\left|N_{F}\right|=\left(\sum \left|C\left(R_{i}\right)\right|+\sum \left|C\left(S_{j}\right)\right|\right)/m$$

$$n_{i}\in N_{R}\;\;\;\;\;\;\;\;\;\;\;n_{j}\in N_{S}$$

where |C(R_i_)| is the number of join candidate pairs arrived from node n_i_ ∈ N_R_. |C(S_j_)| is the number of join candidate pairs arrived from node n_j_ ∈ N_S_.

## 4. Performance Evaluation of IJA

In this chapter, we evaluate the performance of IJA and compare it with typical join strategies such as naïve join, sequential join and centroid join including synopsis strategy. The experiment is mainly measured by the total number of messages incurred for each join strategy because join processing in sensor network is a complex operation due to the distributed nature of the processing and the limited memory at nodes. Other comparisons for performance evaluation and experiments will be included in our future work.

### 4.1. Experiment Environments

The join operation in large scale sensor networks must be processed in a distributed manner. So a single node cannot buffer all the data needed to be joined for most queries. Therefore, for experiments in this work, we performed the same simulation experiments as the synopsis strategy [7] done for comparing naïve join, sequential join, centroid join and also including synopsis strategy. In case of the number of sensor node, this experiment has done with 10,000 sensor nodes uniformly placed in a 100 × 100 grid. Each grid contains one sensor node located at the center of the grid. The regions R and S are located at the bottom-right and bottom-left corners of the network region, respectively, each covering 870 sensor nodes. Table *R* in region R consists of 2,000 pairs, while *S* in region S consists of 1,000 pairs. They are uniformly distributed in regions R and S. For communication cost, we set a message size of 40 bytes, which is equal to the size of a data pair. A pair in the join result is 80 bytes since it is a concatenation of two data pairs. The messages for synchronization and coordination among the sensors are negligible compared to the data traffic for communication caused by large tables. Further, for simplifying analysis, we assume that no failure for sending and receiving messages among nodes.

### 4.2. Performance Evaluation

We first varied the join selectivity and the synopsis selectivity for synopsis strategy. Join selectivity δ is defined as |*R* ∞ *S*| / (|*R*| × |*S*|). The join attribute values are uniformly distributed within the domain of the attribute.

**Figure 3.**
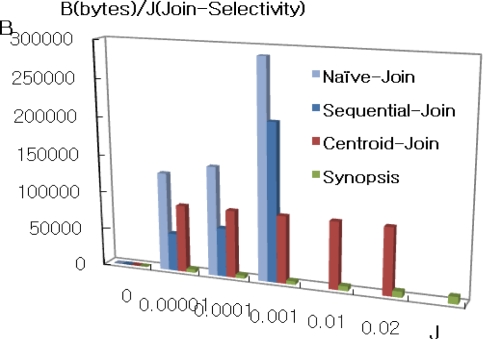
Impact of selectivity.

**Figure 4.**
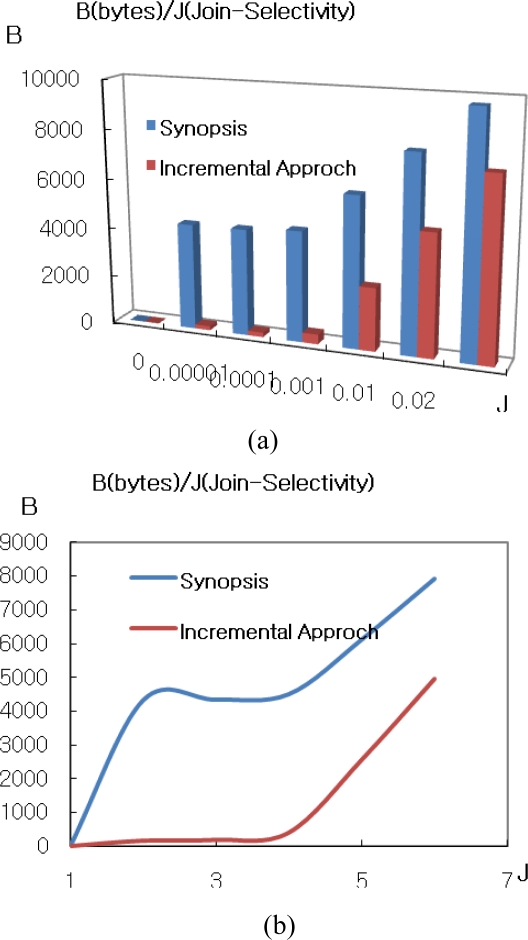
Comparison IJA to synopsis algorithm. **(a)** Comparison synopsis with IJA. **(b)** Magnification with join selectivity <= 0.02.

Figure 3 shows the total communication cost for different join selectivities while keeping the memory capacity and synopsis size fixed at 250 × 40 bytes and 10 bytes respectively. As shown in the Figure, naïve join performs worse than all others due to the high cost of routing *S* in region S to all nodes in N_R_. In addition, sequential join performs worse than centroid join and synopsis as well. Therefore we exclude them from when join selectivity is greater than 0.01. Synopsis strategy is lower than others and outperforms because non-candidate pairs can be determined in the synopsis join state, and only a small portion of data are transmitted during the final join. However, IJA performs than all algorithms though not to be shown in the Figure 3. Therefore Figure 4(a) shows the comparison synopsis algorithm to incremental join algorithm.

**Figure 5.**
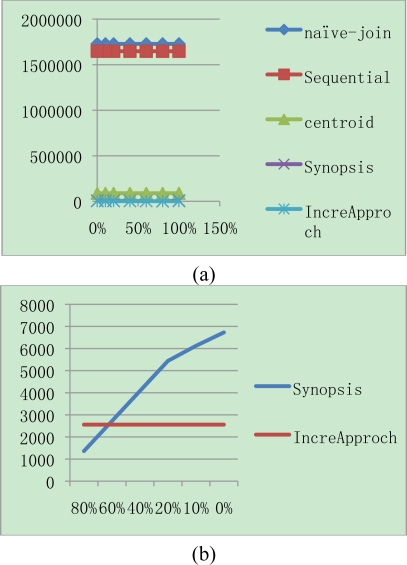
Cases for varied synopsis rates. **(a)** Communication cost as changing synopsis rates from 0% to 100%. **(b)** Comparison IJA with synopsis algorithm.

Figure 4(b) shows the enlargement of lower selectivity than 0.02 in the axis of Figure 4(a). For that case, synopsis has lower communication cost than IJA. This is because synopsis strategy has both join selectivity and synopsis selectivity parameters which have a strong effect on communication cost. For the experiment, the rate for synopsis in this case is fixed with 0.01. In case of synopsis rate variation, it is shown in Figure 5.

We can see the result that the more replicated data are existed, the more the synopsis algorithm is efficient. In spite of that fact, the IJA is more efficient than synopsis methodology under 60% of replicated data. In the environment for above 60% of replicated data, it is unrealistic case. Therefore, the suggested IJA is appropriate for the algorithm in sensor network environment to integrate data.

## 4. Conclusions

Sensor networks have been adopted in various scientific and commercial applications. Gathering data from sensors is achieved by modeling it as a distributed database where sensor readings are collected and processed using queries. Sensor nodes are generally highly constrained, in particular regarding their energy and memory resources. While simple queries such as SELECT and AGGREGATE queries in wireless sensor networks have been addressed in the literature, the processing of join queries in sensor networks remain to be investigated. Previous approaches have either assumed that the join processing nodes have sufficient memory to buffer the subset of the join relations assigned to them, or that the amount of available memory at nodes is known in advance.

Therefore, in this paper including these assumptions, we describe an Incremental Join Algorithm (IJA) in Sensor Networks to reduce the overhead caused by moving a join pair to the final join node or minimize the communication cost that is the main consumer of the battery when processing the distributed queries in sensor network environments. To evaluate the experiments, we compare the IJA with the typical algorithms. including the synopsis algorithm which is a representative strategy in sensor network to process queries. We also show the result of comparisons. In case of under join selectivity 0.01, typical join algorithms, such as naïve, sequence and centroid join, perform worse than synopsis and IJA algorithms.

Despite having better synopsis performance compared to a typical join algorithms, IJA performs better than the synopsis algorithm in conditions with above 60% synopsis. As future work, we will vary and study the parameters such as network density, node memory capacity and synopsis size, including communication cost.

## References

[1] Coman A., Nascimento M.A. A distributed Algorithm for Joins in Sensor Networks.

[2] Mainaring A., Culler D., Plastre J., Szewczyk R., Anderson J. Wireless Sensor Networks for Habitat Monitoring.

[3] Estrin D., Govindan R., Heidemann J.S., Kumar S. Next Century Challenges: Scalable Coordination in Sensor Networks.

[4] Estrin D., Govindan R., Heidemann J.S. (2000). Embedding the internet: Introduction. Commun. ACM.

[5] Bonnet P., Gehrke J., Seshadri P. Towards Sensor Database Systems.

[6] Madden S., Franklin M.J., Hellerstein J.M., Hong W. TAG: A Tiny AGregation Service for *ad-hoc* Sensor Networks.

[7] Yu H., Lim E., Zhang J. In-network Join Processing for Sensor Networks.

[8] Gehrke J., Madden S. (2004). Query processing in sensor networks. Pervasive Comput.

[9] Coman A., Nascimento M., Sander J. On Join Location in Sensor Networks.

[10] Yao Y., Gehrke J.E. (2002). The cougar approach to in-network query processing in sensor networks. SIGMOD Record.

[11] Chowdhary V., Gupta H. Communication-Efficient Implementation of Join in Sensor Network.

[12] Yao Y, Gehrke J. Query Processing for Sensor Networks.

[13] Karp B, Kung M.J. GPSR: Greedy Perimeter Statelss Routing for Wireless Networks.

[14] Sun J.Z. An Energy-Efficient Query Processing Algorithm for Wireless Sensor Networks.

[15] Zhang Z., Gao X.F., Zhang X.F., Wu W.L., Xiong H. Three Approximation Algorithms for Energy-Efficient Query Dissemination in Sensor Database System.

